# Adult Spinal Cord Radial Glia Display a Unique Progenitor Phenotype

**DOI:** 10.1371/journal.pone.0024538

**Published:** 2011-09-12

**Authors:** Audrey Petit, Ashley D. Sanders, Timothy E. Kennedy, Wolfram Tetzlaff, Katie J. Glattfelder, Rachel A. Dalley, Ralph B. Puchalski, Allan R. Jones, A. Jane Roskams

**Affiliations:** 1 Department of Zoology, Life Sciences Institute and International Collaboration On Repair Discoveries (iCORD), University of British Columbia, Vancouver, British Columbia, Canada; 2 Department of Neurology and Neurosurgery, Montreal Neurological Institute, McGill University, Montreal, Quebec, Canada; 3 Allen Institute for Brain Science, Seattle, Washington, United States of America; University of Medicine and Dentistry of New Jersey, United States of America

## Abstract

Radial glia (RG) are primarily embryonic neuroglial progenitors that express Brain Lipid Binding Protein (*Blbp* a.k.a. *Fabp7*) and Glial Fibrillary Acidic Protein (*Gfap*). We used these transcripts to demarcate the distribution of spinal cord radial glia (SCRG) and screen for SCRG gene expression in the Allen Spinal Cord Atlas (ASCA). We reveal that neonatal and adult SCRG are anchored in a non-ventricular niche at the spinal cord (SC) pial boundary, and express a “signature” subset of 122 genes, many of which are shared with “classic” neural stem cells (NSCs) of the subventricular zone (SVZ) and SC central canal (CC). A core expressed gene set shared between SCRG and progenitors of the SVZ and CC is particularly enriched in genes associated with human disease. Visualizing SCRG in a Fabp7-EGFP reporter mouse reveals an extensive population of SCRG that extend processes around the SC boundary and inwardly (through) the SC white matter (WM), whose abundance increases in a gradient from cervical to lumbar SC. Confocal analysis of multiple NSC-enriched proteins reveals that postnatal SCRG are a discrete and heterogeneous potential progenitor population that become activated by multiple SC lesions, and that CC progenitors are also more heterogeneous than previously appreciated. Gene ontology analysis highlights potentially unique regulatory pathways that may be further manipulated in SCRG to enhance repair in the context of injury and SC disease.

## Introduction

Although the cellular neuroanatomy of the SC is well established, we have little understanding of the distinct molecular expression patterns that underpin the functional heterogeneity of its constituent cells. This is particularly true for SC progenitors and neural stem cells (NSCs). The main progenitor identified in the adult SC resides in the ependymal and subependymal layers of the central canal (CC) ventricular zone. CC progenitors express GFAP, BLBP, nestin, SOX2 (Sex determining region Y (SRY)-box 2) and vimentin, and demonstrate NSC characteristics *in vivo* and *in vitro* [1], [2], [3]. The embryonic SC, however, also contains RG similar to the early neural progenitors that generate the majority of neurons and glia throughout the developing central nervous system (CNS) [4], [5], [6]. Embryonic SCRG span the neural tube, with soma located at the ventricular lumen and processes enriched in nestin, vimentin, BLBP and the glial high affinity glutamate transporter (GLAST) terminating at the pial surface. SCRG form a scaffold for the outward migration of newborn neurons that populate the gray matter (GM) [7], [8], [9], [10], after which their soma relocate to the sub-pial edge of the SC (by embryonic day E20), and retract their processes before terminally differentiating into astrocytes (post natal day 15; PND 15) [8], [11]. This astrocytic differentiation is defined by increased GFAP expression and the loss of GLAST, BLBP, vimentin and nestin [10], [12].

In the adult brain, a subset of RG become NSC of the subventricular zone (SVZ) and contribute to ongoing neurogenesis [6], [13], but whether some adult RG might also persist and maintain progenitor activity in the SC is not known. Recently, radially-arrayed WM GFAP^+^ cells have been reported to become mitotic and up-regulate developmental gene expression (e.g. nestin, BLBP, vimentin) following autoimmune demyelination or contusive SC injury [14], [15], [16], [17], concurrent with an accumulation of oligodendrocyte precursors in the sub-pial region [18]. It is thus possible that a secondary, uncharacterized, neural progenitor population resides in the sub-pial WM of the adult SC, whose molecular identity has yet to be established.

The ASCA is an interactive database of >17,000 genes expressed at two distinct developmental stages in the mouse SC that serves as a resource to test for unique and collective expression patterns that enhance our understanding of the molecular identity of neuronal and non-neuronal cells in the SC. Here, we mined the dataset of the ASCA to establish a molecular signature for genes enriched in post-natal SCRG at an immature (plastic, PND 4) and at a more mature (fixed, PND 56) state [19] in order to reveal potential differences between established and novel SC and CNS progenitors. To do this, we used the Anatomic Gene Expression Atlas (AGEA; Allen Institute) tool to delineate collective gene expression patterns within different CNS progenitor niches *in vivo*, to discover potentially novel pathways that may differentially regulate multiple CNS progenitors. We show that both developing and adult SCRG express genes from pathways that regulate proliferation, differentiation and migration in embryonic RG and adult NSC. Our analysis suggests SCRG may have a more extensive *in vivo* potential than previously appreciated, and reveals distinct pathways that may regulate their activation.

The heterogeneity of SCRG as a potentially novel adult SC progenitor population was further established using confocal microscopy to probe for their co-expression of key NSC proteins (BLBP, vimentin, SOX2, nestin). Adult SCRG respond to both contusion and demyelinating lesions by becoming proliferative, up-regulating NSC gene expression, and serving as a structural scaffold for inwardly-migrating cells. These data indicate that in addition to the established progenitors of the CC, a subpopulation of SCRG likely represent a distinct WM progenitor pool, enriched in novel pathways that may be critical in activating them to participate in SC repair.

## Results

### ASCA Reveals the Progenitor Expression Signature of SCRG

**Figure 1:**
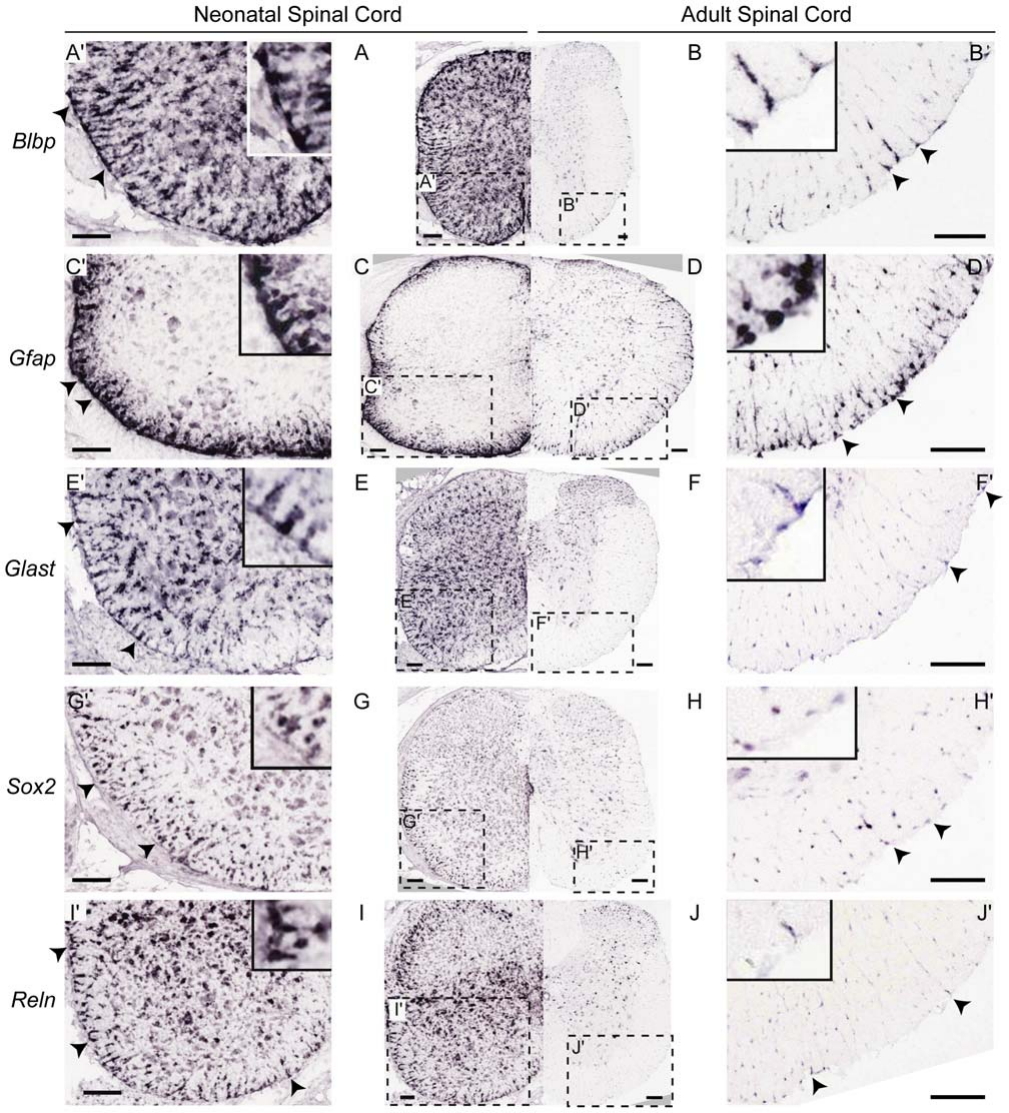
Radial Glia at the Pial Boundary of the Neonatal and Adult Spinal Cord. *In situ* hybridization images from the ASCA show that both neonatal (PND 4) and adult (PND 56) SCRG (arrowheads on soma) express genes commonly associated with embryonic RG. (A, B) *Blbp* (a.k.a. *Fabp7*), (C, D) *Gfap*, (E, F) *Glast*, (G, H) *Sox2* and (I, J) *Reln*. In all cases, SCRG are prominent in the neonatal SC (A, C, E, G & I), and less abundant in the adult (B, D, F, H & J). The boxed areas correspond to the magnified images (A′–J′) and inserts show high magnification of SCRG. Reln: Reelin. Scale bars: 100 µm.

To begin to investigate the cellular heterogeneity of gene expression in SC progenitors, we used combinations of classic RG genes (e.g. *Blbp*, *Glast*) to map the presence and distribution of progenitors of the CC and putative SCRG at PND 4, based on their distinct morphology and expression pattern (Fig. 1A–J). This was used as a guide to blindly score genome-wide SC *in situ* hybridization (ISH) patterns at both PND 4 and PND 56, to produce an RG-specific gene expression dataset that was then augmented by additionally assessing SCRG for known genes enriched in NSCs that may have been missed in the original screen. SCRG gene expression was re-confirmed at multiple cervical-lumbar SC levels before being scored positive. This resulted in the identification of 122 genes that are distinctly expressed in SCRG in the neonatal (PND 4) and/or adult (PND 56) SC (Table S1). SCRG are distributed throughout the presumptive WM in the neonate; however, in the adult SC they appear more heavily clustered in the pial and sub-pial ventral-lateral margins of the WM (Fig. 1), and project processes inward toward the GM and CC.

The gene set identified in both adult and neonatal SCRG includes many established genes commonly expressed in neurogenic embryonic cortical RG, and adult CNS progenitors. For example, *Blbp* (Fig. 1A–B), *Glast* (Fig. 1E–F) and reelin (Fig. 1I–J) are highly expressed in sub-pial RG, even in the adult SC when RG are thought to have all differentiated into astrocytes [11], [20]. Adult NSC genes *Gfap* (Fig. 1C–D) and *Sox2* (Fig. 1G–H) are also expressed and retained in adult SCRG.

### Adult SCRG Express Neural Progenitor Genes

**Figure 2 pone-0024538-g002:**
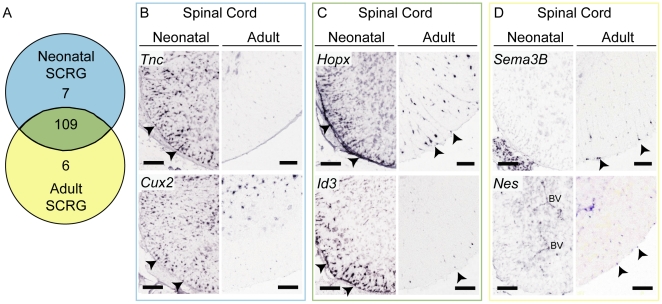
Spinal Cord Radial Glia Shift their Expression of Functionally Important Genes from Neonatal to Adult. (A) Venn diagram indicates the shift in SCRG gene expression during maturation. 7/122 SCRG genes are exclusive to neonate, 109/122 are expressed in both the neonatal and adult SCRG, and 6/122 genes are detected only in adult RG. (B–D) ASCA ISH images of genes expressed in SCRG (arrowheads) at both time points. (B) *Tnc* and *Cux2* are present only in the neonatal, (C) *Hopx* (a.k.a. *Hod*) and *Id3* are present in both neonatal and adult SCRG, while (D) *Sema3B* and *Nes* are exclusive to adult SCRG. Hopx: HOP homeobox; Tnc: tenascin C; Cux2: cut-like homeobox 2; Nes: nestin. Scale bars: 100 µm.

During our initial analysis, it was clear that SCRG are prominent throughout the neonatal SC and become more restricted in the adult, changing gene expression significantly during this time. To investigate this further, each ISH pattern of the 122 genes in the SCRG gene set was closely examined at PND 4 and PND 56, and the range of expression in SCRG was assigned a score from 0 to 3 at both time points (examples in Fig. S1 & Table S1). This analysis revealed that 109/122 genes (89%) are expressed by both neonatal and adult SCRG, although many are reduced in relative expression level and frequency in adult SCRG (Fig. 2A). Many of the genes that persist are implicated in NSC regulation, like HOP homeobox and *Id3* (Inhibitor of DNA binding 3) (Fig. 2C). Only a small subset (7/122 genes, 6%) are exclusively expressed in neonatal (and not adult) SCRG, including NSC regulatory genes like tenascin C and Cut-like 2 (Fig. 2B). An additional subset comprising 6 genes (5%) are expressed exclusively in adult SCRG, including developmentally important genes like nestin and semaphorin 3B (Sema3B) (Fig. 2D). The ontological categories represented by the SCRG gene set were analyzed according to their developmental shift in expression pattern (Table 1). This analysis revealed that many genes identified have established roles in proliferation, differentiation and adhesion or migration, and continue to be expressed in adult SCRG. As SCRG mature from a neonatal plastic to a more fixed adult state, there is a distinct shift in expression of genes that regulate progenitor interaction with extracellular matrix (ECM), and cell-cell communication (channels and transport molecules).

**Table 1:** Transcriptional Profile of SCRG Highlights a Progenitor Cell Phenotype.

Functions	Expression	List of Genes in Each Gene Ontology						
	Neonatal only	*Ncan*	*Tnc*					
	Adult only	*Aebp1*	*Msx1*	*Sparc*				
Adhesion	Both time	*Agt*	Apc**	*Apoe*	Ascl1**	*Bmpr1a*	Ccnd1**	*Cdn9*
& Migration	points	*Clu*	*Col14a1*	Egfr**	*Fabp7*	*Fgfr3*	Gab1**	*Gja1*
22.13%		*Hexb*	*Hey2*	*Hopx*	*Id3*	*Id4*	MapK1**	MapK3**
		*Nsdhl*	Pax6**	*Qk*	*Reln*	*Slc1a2*	*Slc1a3*	*Sox2*
		*Spr1*	*Stx2*					
	Neonatal only	*Fxyd6*	*Gnptab*	*Kcnmb4*	*Slc6a11*			
Channels and	Adult only	*No unique genes*						
Transport	Both time	*Abca1*	*Apoe*	*Aqp4*	*Atp1a2*	*Dbi*	*Fabp5*	
Molecules	points	*Fabp7*	*Gja1*	*Kcnj10*	*Mlc1*	*Pcna*	*Qk*	
18.85%		*Sfxn5*	*Slc1a2*	*Slc1a3*	*Slc39a12*	*Stx2*	*Tpcn1*	*Tst*
	Neonatal only	*Tnc*						
	Adult only	*Lgi4*	*Msx1*	*Sema3b*				
	Both time	*Agt*	Alcam**	Aldh1a1**	Apc**	*Apoe*	*Ascl1*	*Bmpr1a*
Development	points	*Clu*	Egfr**	*Fabp7*	*Fgfr3*	Gab1**	*Gja1*	*Gjb6*
34.43%		*Hexb*	*Hey2*	*Hopx*	*Id3*	*Id4*	MapK1**	MapK3**
		Meis2**	Msi2**	Myc**	*Nsdhl*	Pax6**	Pou5f1**	*Qk*
		*Reln*	Sall4**	*Slc1a2*	*Slc1a3*	Smad1**	*Sox2*	*S1pr1*
		*Stx2*	Tcl1**	Tob2**				
	Neonatal only	*No unique gene*						
Differentiation	Adult only	*No unique gene*						
12.30%	Both time	Alcam**	Apc**	Ascl1**	*Bmpr1a*	Egfr**	*Hopx*	*Id4*
	Points	MapK1**	MapK3**	Msi2**	Pax6**	Pou5f1**	Smad1**	*Sox2*
		Tcl1**						
Extracellular	Neonatal only	*Ncan*	*Tnc*					
Matrix Molecules	Adult only	*Sparc*						
6.56%	Both time points	*Col14a1*	*Col23a1*	*Reln*	*Slc1a3*	*Sparcl1*		
	Neonatal only	*No unique gene*						
Proliferation	Adult only	*Sparc*						
18.03%	Both time	*Agt*	Apc**	*Apoe*	*Bmpr1a*	*Clu*	Egfr**	*Fabp7*
	points	*Fgfr3*	Gjb6**	*Hey2*	*Hopx*	*Id4*	MapK1**	*II6st*
		MapK3**	Myc**	Pax6**	*Pcna*	Smad1**	*Sox2*	*S1pr1*
	Neonatal only	*Fxyd6*	*Ncan*	*Tnc*				
	Adult only	*Aebp1*	*Lgi4*	*Msx1*	*Sema3b*	*Sparc*		
Signalling	Both time	Alcam**	*Agt*	Apc**	*Apoe*	Ascl1**	*Bmpr1a*	Ccnd1**
Molecules	points	*Clu*	*Col14a1*	Crtap**	*Cst3*	Dbi *	Egfr**	Errfi1**
37.70%		Fabp5 *	Fabp7 *	*Fgfr3*	Fkbp9**	Gab1**	*Hexb*	*Il6st*
		*Lcat*	Lgr5**	*Lrrc55*	*Lrrn1*	MapK1**	MapK3**	Myc**
		*Myoc*	Pax6**	*P4ha3*	*Plat*	Prom1**	Rcn1**	*Reln*
		Scd3 *	Smad1**	*Sparcl1*				
	Neonatal only	*Cux2*						
Transcription	Adult only	*Aebp1*	*Msx1*					
Factors	Both time	Ascl1**	Ezh2**	*Hey2*	*Hopx*	*Id3*	MapK1**	*Id4*
18.85%	points	MapK3**	Maz**	Meis2**	Myc**	Mycbp**	Pou5f1**	Pax6**
		Sall4**	Smad1**	*Sox2*	*S1pr1*	Tead2**	Utf1**	

Ontological categories represented by the SCRG gene set reveal gene groups that are important in NSC regulation. The groups identified include pathways important for cell adhesion and migration, differentiation, proliferation, many signaling molecules and transcription factors. The PPAR signaling pathway is significantly represented (p = 0.036) within the SCRG gene list (genes marked by single asterisks). Percents indicate the number of genes in the ontology grouping, compared with the total number of genes. Genes marked with double asterisks were identified by data mining the literature and databases for NSC genes, and performing additional analysis of expression within RG.

### Neonatal and Adult SCRG Retain a Neural Progenitor Morphology and Expression Profile

**Figure 3:**
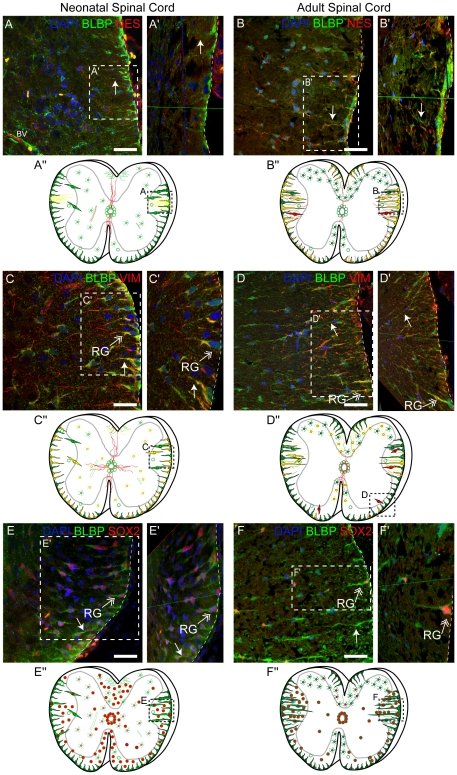
Spinal Cord Radial Glia Heterogeneity is Highlighted by the Co- Expression of Neural Stem Cell Proteins in the Neonatal and Adult Spinal Cord. (A, C, E) In the neonatal (PND 5) SC, confocal imaging shows that BLBP^+^ (green) SCRG extend processes (arrows) from the marginal edge of the SC and through the WM. These cells (A) rarely co-express NES (red), but distinct subpopulations (B) robustly co-express VIM (red) and (E) nuclear SOX2 (red). Boxed areas outline higher magnifications (A′–F′) of the z-stacks, rotated on a 3D plane to highlight the anchored SCRG cytoarchitecure at the pial boundary. (B, D, E) In the adult (PND 75) SC, the BLBP^+^ (green) SCRG are less abundant, but their processes display enhanced expression of (B) NES (red) and (D) VIM (red). (F) SCRG nuclei remain at the sub-pial edge of the SC, where rare subpopulations retain expression of SOX2 (red). (A″–F″) Distribution of progenitor marker expression is depicted in schematics of SC cross-sections, and highlight the shifting cytoarchitecture of the SCRG and CC progenitors of the neonatal and adult SC. Immuno-positive multipolar cells and BV are included. Arrow: SCRG process; double arrow: SCRG; dotted line: marginal edge of SC; BV: blood vessels. Scale bars: 50 µm.

To further test if the protein products of these expressed NSC genes may be co-expressed in a way that reveals greater heterogeneity in the potential of SCRG than currently appreciated, we used immunofluorescent detection to see if of some of the key NSC genes - vimentin, SOX2, nestin – were co-expressed with BLBP. In the neonate, BLBP^+^ SCRG are aligned along the outer edge of the developing WM, and are most easily detected in the ventrolateral regions and extend short, thick processes towards the GM (Fig. 3A, C, E). Although nestin is commonly used to identify RG or NSCs, it is not expressed by the majority of neonatal SCRG, but a few nestin^+^ processes extend from the lateral SC into the WM and co-express BLBP and vimentin (Fig. 3C and Fig. S3A). In contrast, adult SCRG co-express BLBP (Fig. 3 B, D, F) and vimentin (Fig. 3 D), and many co-express nestin (Fig. 3B and Fig. S3B). Neonatal SCRG niches are highly proliferative, based on their PCNA expression (Fig. S3C), and proliferation zones appear clustered at the ventrolateral WM margin, where subpopulations of BLBP^+^ SCRG co-express SOX2 (Fig. 3E–F). In both neonate and adult, SOX2^+^ cells are found in two major SC sites - at the CC and all around the WM pial boundary. Detailed schematics of thoracic SC sections depict the precise distribution of immuno-positive cells for these progenitor markers, and map the developing cytoarchitecture of SCRG from neonate to adult (Fig. 3A″–F″; Fig. S3A″–D″). These schema indicate that SCRG expressing progenitor markers are aligned at the outer edge of the developing WM in the neonate, but in the adult they are clustered more in the ventrolateral margins, extending short and thick processes towards the GM. This combination of NSC antigens highlight a distinctive and heterogeneous expression pattern in both neonate and adult SCRG that further suggest the sub-pial niche may contain heterogeneous progenitors. Their differential expression and distribution also indicate there could be some key similarities and differences between SCRG and CC progenitors.

### SCRG Gene Expression is Distinct from Ventricular CNS Neural Progenitors

**Figure 4 pone-0024538-g004:**
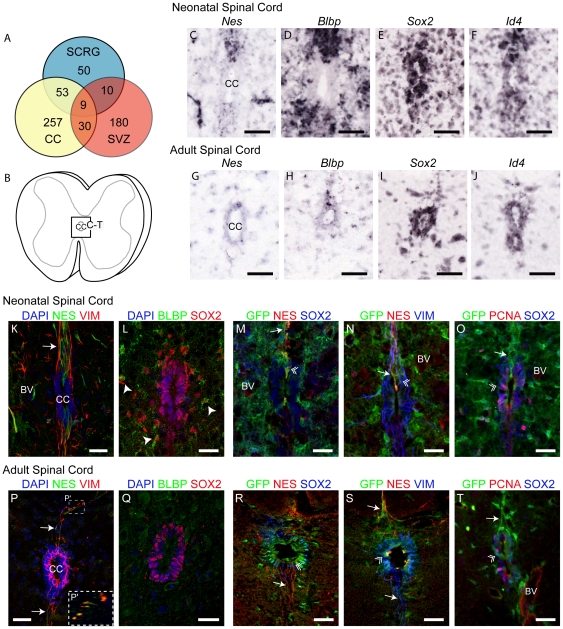
Progenitor Gene Expression in Neonatal and Adult Central Canal. (A) Venn diagram illustrating the number of genes that overlap (or are unique) in expression between CC, SCRG and SVZ. (B) Schematic of SC cross-section detailing the position of the CC progenitor niche. (C–J) *ISH* images from the ASCA show the shifts in expression of key neural progenitor genes shared with SCRG in the (C–F) neonatal and (G–J) adult CC: (C, G) *Nes*, (D, H) *Blbp*, (E, L) *Sox2* and (F, J) *Id4*. (K–T) Confocal z-stack images of neural progenitor proteins in the CC of (K, L) neonatal and (P, Q) adult wild-type mice, and (M–O) neonatal and (R–T) adult Fabp7 (BLBP)-EGFP transgenic reporter mice (M–O) neonatal and (R–T) adult CC (where all reporter^+^ cells are GFP^+^). In the (K, M, N) neonatal CC, NES^+^ processes emanate dorso-ventrally from the CC, some of which co-express VIM and are more abundant than in the (P, R, S) adult CC. In the (L, M, O) neonatal SC, SOX2^+^ nuclei are clustered within and around (arrowheads in L) the CC some of which co-express BLBP or GFP (green), but (Q, R, T) in the adult, become tightly restricted to the ependymal and subependymal layers. (O) Few PCNA^+^ (red) cells are localized around the CC in both the neonatal and (T) the adult, some of which are also GFP^+^ (double arrowheads). (P) Boxed area corresponds to magnified inset (P′). CC: Central canal; arrowhead: soma; arrow: process; double arrowhead: triple-positive cell; dotted line: marginal edge of SC; VIM: Vimentin; BV: blood vessel. Scale bars: 50 µm.

The collective gene expression and phenotypic analysis (Figs. 1–3) suggests that two distinct neurogenic niches may exist in the SC (CC and SCRG), containing cells that may have different potentials and different modes of regulation. To explore this further, we designed a cross-comparative niche expression analysis to test if other potential unknown NSC genes may be expressed in SCRG that are also found in NSC of the CC and cortical SVZ. CC gene expression was scored from the ASCA in a similar manner described for SCRG; where both anatomical location (within the CC) and gene expression coincident with the CC progenitor markers nestin (Fig. 4C, G) and *Sox2* (Fig. 4E, I) were used to identify 349 CC-enriched genes. These genes also fitted into distinct expression groups, where some (e.g. *BLBP*, nestin) were more segregated to dorsal and ventral poles of the CC (Fig. 4C–D, G–H), others (*Sox2*, *Id4*) were evenly distributed and equally represented in neonate and adult (Fig. 4E–F, I–J). SVZ-enriched gene expression was assayed using the AGEA tool by sampling three different sites in the SVZ rostro-caudal axis bilaterally to obtain a candidate gene list. Genes expressed in adjacent cortical regions were then negatively subtracted to yield 229 genes enriched in the SVZ. The cross-comparison showed that SCRG share 62 genes (51% of the SCRG gene set) with the CC and 39 genes (32%) with the SVZ (Fig. 4A). Of these genes, 9 (7%) are expressed by progenitors in all three niches (functions of these genes are included in Table S2), some of which have distinct expression patterns and roles in early brain or tissue development. This core gene set includes the embryonic stem cell regulatory transcription factor *Sox2*, a critical Notch-pathway transcription factor (*Ascl1*), along with transcription factors implicated in stem cell maintenance (*Lxn*) and differentiation of myelinating cells (*Id4*) [21], [22], [23]. Additional genes in this dataset (*Mlc1*, *Gja1*, *Cd63*) are human disease genes that had not previously been localized to CNS progenitor niches.

### CC Progenitors are dynamic and heterogeneous

These distinct expression patterns suggest significant variation in both SC progenitor niches, and indicate that the CC may be a complex progenitor zone containing subpopulations of progenitors with different potentials. To further test this, we used immunofluorescent detection of some of the “shared” NSC gene products - nestin, vimentin, *Sox2* - with the mitotic marker (PCNA) to further map the compartmentalization of the CC. This analysis was further enhanced by using triple immunofluorescence, for NSC markers in BLBP-expressing putative progenitors of the Fabp7-EGFP mouse (Fig. 4M–O, R–T). In the neonate, progenitors enriched in nestin are clustered at the dorsal and ventral poles of the CC (Fig. 4M–N), extending processes dorsally and ventrally that occasionally co-express vimentin, and BLBP, or GFP (Fig. 4K, M–N). These dorso-ventral nestin-expressing cells are rarer in the adult CC (Fig. 4P, R–S). Subsets of SOX2- expressing cells within and around the neonatal CC co-express PCNA or BLBP (Fig. 4L–M, O), but the anatomical complexity of these cells is more clearly visualized when GFP is detected in cells driving the Fabp7 promoter (Fig. 4M–O). The neonatal CC contains some BLBP^+^ cells (Fig. 4L, Q), but GFP detection indicates that most are clustered around the CC, and are likely to represent developing astrocytes, whereas GFP^+^ cells are more restricted to the CC in the adult. A significant population of SOX2-expressing cells are detected in both neonatal and adult CC, with some adult cells retaining co-expression of BLBP (GFP) but rarely nestin (Fig. 4L–M, O, Q–R, T). Subpopulations of SOX2^+^ cells also express BLBP/GFP and extend nestin^+^/vimentin^+^ processes that terminate abruptly at the GM-WM interface (Fig. 4M–N, P, R–S). These data indicate that CC progenitors are a highly heterogeneous group of cells that are distinct in form, function, and regulation, from both SVZ progenitors and SCRG.

### Fabp7-EGFP-Expressing Adult SCRG encircle the SC and are phenotypically heterogeneous

Although the immunofluorescent detection of BLBP putative SC progenitors has allowed us to identify a novel SCRG population where BLBP is co-expressed with NSC antigens, it is possible that similar to the CC, (Fig. 4M–O, R–T) antigen detection is limited in its ability to detect SCRG that have down-regulated BLBP.

**Figure 5 pone-0024538-g005:**
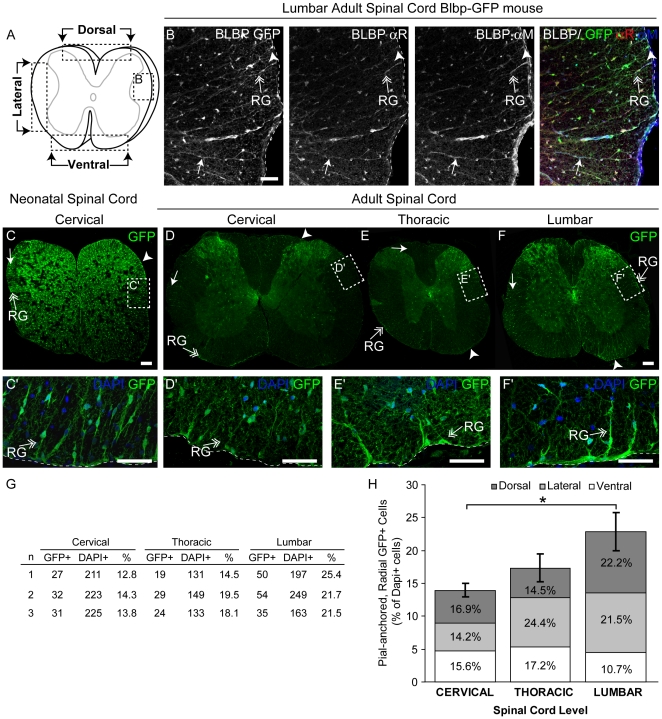
Radial Glia Are Distributed in an Increasing Cervical-to-Lumbar Gradient in the Spinal Cord of the Adult Fabp7-EGFP Reporter Mouse. (A) Schematic of SC cross-section detailing the position of the dorsal, lateral and ventral areas assayed in B–F, with arrowheads marking the anatomical boundaries for quantification. (B) Expression of eGFP was detectable, but enhanced by a GFP antibody, that confirms GFP co-localization with both rabbit and mouse anti-BLBP antibodies. (B–F) Confocal z-stack images of (C) neonatal (PND 5; cervical level) SC and (B, D–F) adult SC (P56; cervical, thoracic and lumbar levels) Fabp7-EGFP mice. (C) In the neonate, GFP^+^ SCRG are abundant all around the SC. (D–F) In the adult SC GFP illuminates the cytoarchitecture of the processes and soma of pial RG (double arrow) and reveals that (G, H) GFP^+^ SCRG significantly increase in frequency from cervical to thoracic and lumbar levels (p<0.05), and also shift in their relative distribution in dorsal, lateral and ventral SC at different levels. Boxed areas outline magnified regions (C′, D′–F′) of the z-stack. Arrowhead: soma; arrow: process; double arrow: SCRG; dotted line: marginal edge of SC. Scale bars: 50 µm.

In contrast, the accumulation of GFP in progenitors containing transgenic reporter genes frequently provides a means to better visualize rarer cell subpopulations in the CNS, so we next tested for GFP distribution in SCRG of the Fabp7-EGFP reporter mouse. This approach clearly allowed us to more readily image the distribution of all SCRG (regardless of their BLBP expression level), and highlights the complex cytoarchitecture and distribution of all SCRG (that were not detectable by BLBP IF alone). We first confirmed, using two different Blbp antibodies, that GFP is specifically and exclusively expressed by Blbp^+^ cells in the lateral WM of the SC (Fig. 5A–B). The distribution of BLBP/GFP in the neonate (Fig. 5C) shows SCRG all around the sub-pial developing WM (similar to ISH and IF; Figs. 1–3), in addition to the extensive neonatal production of newborn astrocytes. In the adult, GFP^+^ SCRG persist and form a scaffold throughout the SC WM, along the dorsal-ventral axis (dorsal, lateral, and ventral) (Fig. 5D–F) at three rostral-caudal levels (cervical, thoracic, and lumbar). The expression of GFP^+^ throughout SCRG, also made it easier to visualize a clear caudal-to-rostral gradient of SCRG (Fig. 5D–H), where 14.0±1.0% of the cells along the WM surface are SCRG at cervical levels, 17.3±2.1% in the thoracic, and significantly increase to 22.9±2.9% (p<0.05) in the lumbar SC. The distribution of SCRG also shifts dorsally along this gradient. In the cervical level the percentage of SCRG is similar between the ventral (15.6±0.8%), lateral (14.2±2.5%) and dorsal (16.9±4.0%) areas, in the thoracic they predominate at the lateral regions (24.4±4.1%) and in the lumbar cord most SCRG are present at the dorsal pole (22.2±5.9%) and also in the lateral area (21.5±2.7%) with a lower percentage at the ventral pole (10.7±1.6%).

**Figure 6:**
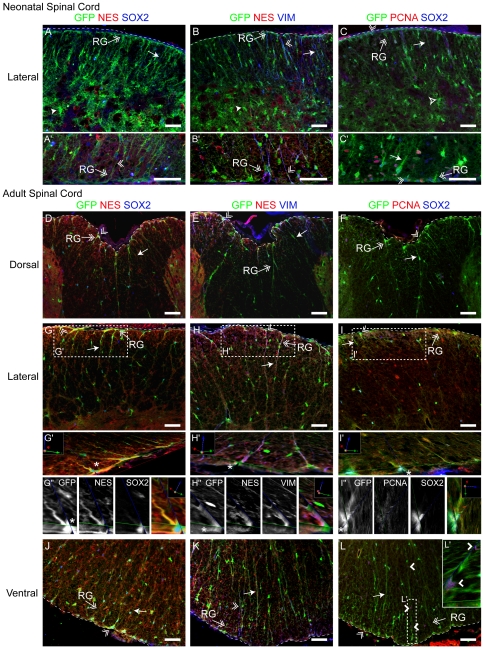
An Extensive Scaffold of Fabp7-EGFP-Expressing Spinal Cord Radial Glia Retain Progenitor-like Morphology and Gene Expression. (A–L) Confocal imaging of the SC of (A–C) neonatal (PND 5; cervical level) and (D–L) adult (P56; lumbar level) Fabp7-EGFP mice has allowed us to reveal a more extensive population of SCRG than detected by immunofluorescence (A–C) BLBP^+^ (green) SCRG processes (arrows) begin at the marginal edge of the SC and extend throughout the WM and up to the astrocyte-rich (arrowhead) GM. Subpopulations of GFP^+^ SCRG co-express (A, C) SOX2 (blue) and (B) VIM robustly (blue) but rarely (A, B) NES (red). Many nuclei contain (C) PCNA (red). (D–L) Although less abundant than in the neonatal SC, the (D–L) GFP^+^ (green) processes of adult SCRG also demonstrate enhanced expression of (E, H, K) VIM (blue) and (D, E, G, H, J, K) NES (red). Their nuclei remain at the sub-pial edge of SC and small subpopulations retain expression of (D, F, G, I, J, L) SOX2 (blue) and (F, I, L) PCNA (red). Boxed areas outline magnified regions (G′–I′, L′) of the z-stack that were rotated and tilted on a 3D plane to best highlight the anchored cell soma and processes at the pial boundary. (G″–I″) Gray scale images of a single SCRG cell (asterisk in G′–I′). Arrowhead: astrocyte; arrow: process; double arrowhead: triple-positive cell; double arrow: SCRG; open arrowhead: cell on SCRG process; dotted line: marginal edge of SC; BV: blood vessels. Scale bars: 50 µm.

In the neonate, GFP^+^ cells are found throughout the developing GM and WM (Fig. 5C, C′; Fig. 6A–C), where the majority of them have the morphology and distribution of developing astrocytes. In contrast, GFP^+^ SCRG are a dense population of cells aligned at the outer edge of the neonatal WM extending processes sideways, parallel to the SC surface, and an inwardly-projecting array of defined, thick processes towards the GM with migratory cells aligned along them (Fig. 5C, C′; Fig. 6A–C). The majority of neonatal GFP^+^ SCRG co-express vimentin (Fig. 6B, B′), and although the majority of neonatal SCRG do not co-express nestin (Fig. 6A–B), a few nestin^+^ processes extend from the lateral edges into the WM and co-express vimentin (Fig. 6B, B′). Moreover, significant subpopulations of neonatal SCRG are dividing (PCNA^+^), and co-express SOX2 (Fig. 6C, C′).

Analysis of the adult Fabp7-EGFP reporter SC confirms that adult SCRG are less abundant in the adult, but, in contrast to earlier data suggesting they are largely clustered in the lateral WM (Figs. 1, 3 and Fig. S3), reveals that they are distributed all around the WM-pial interface, with longer, thinner processes than their neonatal counterparts. With GFP illuminating the cytoarchitecture of all SCRG regardless of their BLBP expression level (Fig. 6 A–L; Fig. S2), it is clear that a significant proportion of adult SCRG co-express vimentin (Fig. 6E, H, H′, H″, K; Fig. S2B), and nestin (Fig. 6D, G, G′, G″, J; Fig. S2A–B) in the lateral (Fig. 6G–I), dorsal (Fig. 6D–F) and ventral WM where long SCRG processes are enriched and extend toward the GM-WM interface. Given their high expression of intermediate filament proteins, it is possible that SCRG may serve as a more structural scaffold in the adult SC, but readily detectable subpopulations of adult GFP^+^ SCRG co-express SOX2 (Fig. 6D, F–G, I–J, L; Fig. S2A, C) and occasionally PCNA (Fig. 6F, I, I′, I″, L; Fig. S2C), indicating some adult SCRG do retain a progenitor phenotype. At all levels, SCRG form inwardly-projecting elongated processes that traverse the WM, with bipolar migratory GFP^+^ cells associated with them (Fig. 6A–L), some of which co-express SOX2- or PCNA (Fig. 6L′).

### SCRG Expand and Transform During the Lesion Response to SC Crush and Autoimmune Encephalomyelitis

**Figure 7:**
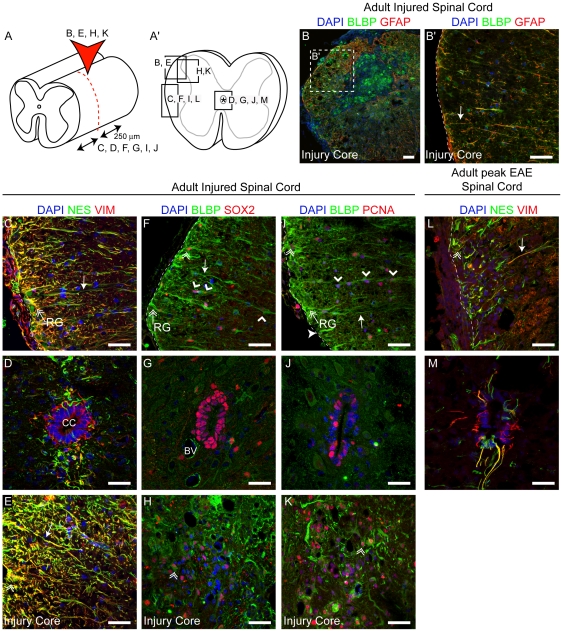
Spinal Cord Radial Glia Expand and Transform Morphologically During the Response to SC Compression and Experimental Autoimmune Encephalomyelitis. Confocal z-stack images of an adult SC reveal similar responses by SCRG and CC progenitors 14 days after a (A) dorsal column compression (red arrowhead), where analysis was performed both (B, B′, E, H, K) at the injury core and (C, D, F, G, I, J) peri-lesion area (≤250 µm rostral or caudal from the site of injury). (A′) Schematic of SC cross-section detailing the position of (B′–M) images (B–B′) After dorsal column compression BLBP^+^/GFAP^+^ SCRG appear disorganized but their processes are readily detected. In the peri-lesion area, SCRG processes appear hypertrophied and strongly express (C–E) NES (green) and VIM (red). Nuclei containing (F–H) SOX2 or (I–K) PCNA are more abundant after lesion in all locations - at the pial boundary, arrayed along BLBP^+^ (green) processes and (G, J) at the CC. (L–M) Analysis of expression following the induction of EAE showed a similar profile of SCRG hypertrophy and CC expansion. CC: Central Canal; arrow: process; double arrowhead: double-positive cell; double arrow: SCRG; open arrowhead: cell on SCRG process; dotted line: marginal edge of SC Scale bars: 100 µm (A), and 50 µm (A′–M).

Endogenous SC progenitors are thought to have the capacity to enhance SC repair in the context of injury, where CC progenitors divide and produce migratory progeny in response to lesion [24]. Having established that SCRG demonstrate progenitor gene expression, we tested if they too respond to injury by shifting in mitotic or morphological state and gene expression in response to a defined forceps compression (Fig. 7A–K), or experimental allergic encephalomyelitis (EAE) (Fig. 7L, M).

Both SCRG (Fig. 7B,F,I) and CC (Fig. 7D,G,J) progenitors divide and up-regulate developmental gene expression close to the injury core and up to 250 µm away (Fig. 7A), at 14 days after SC compression. In the sub-pial zone, BLBP^+^ SCRG processes up-regulate nestin and vimentin, and appear hypertrophied and more abundant (Fig. 7B–C, E), compared with uninjured SC controls (Figs. 3–6; Figs S2, 3). Similarly, nestin^+^/vimentin^+^ dorsal midline processes extending from the CC appear hypertrophied, retracted and less organized in the lesioned SC (Fig. 7D), compared to the control (Fig. 4). Following lesion, cells expressing SOX2 become more abundant in both progenitor zones and throughout the WM (Fig. 7F–G). SOX2^+^ cells are especially concentrated all around the CC and within and arrayed along BLBP^+^ SCRG (Fig. 7F–G). At the site of the injury many sub-pial SOX2^+^ cells co-express BLBP and have a distinct RG morphology (Fig. 7H). Even 14 days after SC compression, there is extensive active mitosis of PCNA^+^ cells throughout the injured SC, many of which are concentrated around the CC, and others are SCRG co-expressing BLBP in the proximity of the lesion (Fig. 7 I–K). Compared to uninjured controls, PCNA^+^ and SOX2^+^ cells often appear in chains aligned along BLBP^+^ SCRG (arrowheads in Fig. 7F, I). Distinct SCRG and CC progenitor responses are also mounted during the peak phase of EAE autoimmune demyelination; marginal SCRG processes become heavily intertwined (Fig. 7L) and nestin^+^/vimentin^+^ processes radiate from all around the CC (Fig. 7M) in contrast to the dorsoventral-restricted projections of the normal SC (Fig. 4 P, P′, R–S).

Collectively, these data indicate that a subpopulation of cells in the marginal WM of the neonatal and adult SC share physical and regulatory characteristics with embryonic RG and adult ventricular NSCs, and may represent a unique and distinct progenitor that functions in a non-ventricular niche.

## Discussion

Here, we report that an extensive framework of SCRG are found throughout the WM of the adult SC, that have a novel *in vivo* expression signature that suggests they may function as a potential progenitor situated at the SC-pial interface. The SCRG gene expression signature includes regulatory pathways that are shared with, and also distinct from, ventricular CNS progenitors, which offers new avenues to exploit and activate SCRG to participate in the repair response following SC injury. In addition, we reveal a complex heterogeneity of progenitor phenotypes within the CC that are distinct from SCRG, and identify a core NSC gene signature comprising several genes associated with human disease that can now be functionally placed within multiple CNS progenitor compartments. Finally, gene expression shifts from neonatal to adult SCRG reveal genes that are up- and down-regulated as the SC matures from a more plastic state to one that is recalcitrant to repair after lesion, and provides pathways to further investigate in developing novel strategies for enhancing recovery from SC lesion [19].

Using established RG genes as a roadmap for characterizing the cell-specific expression profile of SCRG within the 17,000 genes of the ASCA allowed us to perform comparative gene expression analysis of neonatal versus adult SCRG, to identify distinct gene sets that may regulate SCRG phenotype, activation and potential (Figs. 1, 2). Many of the genes continuously expressed in SCRG have established developmental roles in regulating NSC function (*Sox2*, reelin, *Id3*, *Id4*, quaking). Neonatal and adult SCRG are differentially regulated by morphogens (BMPR1a), ECM molecules (tenascin C) and transcription factors (CUX2, S1PR1). In contrast, adult SCRG express genes encoding proteins that direct axon growth and cell migration (semaphorin 3B) and repress mitosis and differentiation (AEBP1, MSX1). Although there is heterogeneity in the expression of RG genes and proteins within SCRG at both ages (by ISH and immunofluorescence analyses), vimentin is always co-expressed with BLBP, as it is in both proliferative embryonic RG and adult cerebellar Bergmann glia [25].

Although elongated cells in the SC WM have usually been classified as “astrocytes,” the gene expression signature reported here widens the gulf between SCRG and mature astrocytes, and indicates SCRG have much more in common with CNS progenitors. GFAP is usually used to identify mature astrocytes, but it is also highly expressed by neural progenitors [6]. Although all SCRG appear to express GFAP, they are highly heterogeneous, and distinct subpopulations co-express the progenitor genes *Blbp*, *nestin* and *vimentin* (Figure 3). Similarly, although nestin is commonly used as a “RG marker” and highly expressed by all embryonic RG [20], it is rarely detected in neonatal, yet readily detected in adult, SCRG (Figs. 2, 3, 4). Thus, postnatal down-regulation of nestin in SCRG is transient, and concurrent with the peak of gliogenesis and myelination, leaving open the possibility that the re-induction of nestin expression in adult SCRG may (as in other NSCs) indicate a shift to a different, adult, progenitor state. In contrast, it is clear that SCRG do not express the “astrocyte genes” *Ntsr2* and *Acsbg1* [26], further indicating that at least some SCRG are clearly distinct from astrocytes. Interestingly, some scattered cells in the neonatal SCRG zone express the astrocyte-specific gene *Aldh1l1*, but the majority of these cells are arranged in chains along processes of SCRG. This supports the proposed existence of an intermediate glial precursor interposed between SCRG and immature astrocytes [10], which use SCRG as a migratory physical framework. While some SCRG genes are indeed transiently expressed by proliferative neonatal astrocytes (*Blbp*, vimentin), several genes associated with embryonic RG function are preserved in adult SCRG and are not expressed by astrocytes (*Sox2*, reelin). Collective analysis of the function of genes expressed by SCRG thus suggests they may be capable of the NSC and migration-guidance functions of embryonic RG, given a conducive environment.

Although many embryonic SCRG clearly do become astrocytes postnatally, genes expressed by RG and NSCs persist in SCRG subpopulations in the adult SC WM, but not in astrocytes. This is most clearly visualized in the SCRG of the Fabp7-EGFP mouse, where GFP illuminates an extensive array of SCRG in the adult SC (Figs. 5, 6). The difference in SCRG abundance between the reporter (Figs. 5–6; Fig. S2), and the ISH (Fig. 1), or IF detection of BLBP (Fig. 3; Fig. S3) is likely due to a lower expression of BLBP in dorsal and ventral SCRG, where some SCRG may have down-regulated BLBP, yet retained GFP throughout their soma and processes. Regardless, it is clear that the previously presumed widespread differentiation of SCRG into astrocytes postnatally [20], may be similarly due to technical limitations in detection, the transient down-regulation of nestin (used as a classic RG marker) postnatally, and a revised understanding of what constitutes NSC, as opposed to astrocyte “markers”. It is also clear that the GFP reporter has revealed a distinct spatial gradient of SCRG, where their incidence increases from the cervical to lumbar SC. The functional implications of this gradient, in terms of structural stability and repair of the SC WM, are not known.

In order to target the activation of distinct progenitors in repair, it is important to identify regulatory genes that are shared, and unique, to NSCs in different compartments. Our trans-niche progenitor expression analysis (Fig. 4) revealed core pathways of NSC regulation enriched in SCRG, that are also conserved in ventricular progenitors of the CC and SVZ. These genes represent regulatory pathways that may serve a common role in progenitors residing in distinct CNS locations. Of the core nine genes shared in all three progenitor niches, some are known NSC regulatory genes (*Sox2*, *Ascl1*, *Lxn*, *Id4*), whereas others (*Mlc1*, *Gja1*, *Cd63*) are human disease genes that now represent candidate regulatory pathways that serve a common role in regulating different CNS progenitors. Of these, *Mlc1* deserves particular attention for its potential function in postnatal progenitors, as mutations in *Mlc1* lead to the chronic demyelinating disorder Megalencephalic leukoencephalopathy with Subcortical Cysts- a disease that features an over-production of neural cells early during the first year of life that results in a paucity of myelin and macrocephaly [27]. Although *Mlc1* function is uncharacterized, its enrichment in all three NSC niches suggests a regulatory role in postnatal progenitor proliferation.

The trans-niche analysis also identified genes that are distinctly distributed between the different CNS progenitor subpopulations, and augment our current understanding of what makes the progenitor zones of the CNS different in both neonate and adult (Figs. 2, 4). The genes shared by SCRG and CC that are not in the SVZ point to conserved regulatory pathways that may be unique to SC progenitors. In addition, the physical separation of the GM CC and WM SCRG niches, coupled with distinct expression profiles, suggests that progenitors with different potentials may be segregated into distinct compartments within the SC. This may enable local signals to regulate their ability to serve different maintenance or repair functions for different GM and WM environments. Several of the genes in common between SCRG and CC regulate mitosis, suggesting that some core conserved mechanisms may regulate SC progenitor proliferation. Conversely, the divergent (unique) gene expression of ECM-related pathways within these progenitor pools, coupled with niches situated in different environments, indicate that local stimuli – in quiescence and after lesion - most likely regulate their potential. Ontological analysis of SCRG gene expression (Table 1) has highlighted pathways that could be targeted to specifically direct adult SCRG into a pro-repair state in the lesioned SC. For example, the Peroxisome Proliferator-Activated Receptor (PPAR) pathway is highly represented in the SCRG dataset and has recently been implicated in plasticity and repair of the adult CNS. The anti-inflammatory and neuroprotective functions attributed to PPAR signaling [28], [29], coupled with their newly-revealed enrichment in SCRG, make this pathway a promising therapeutic target to enhance the reparative capacity of the SC.

Recent SC lesion studies have highlighted the existence of peripherally-situated WM dividing cells that can respond to SC injury and EAE and contribute to the remodeling of the SC [15], [18], [30]. For instance, SC WM “astrocytes” located at the pial surface and extending processes towards the CC significantly up-regulate nestin during the proliferative response to SC contusion [31]. Here we show, in two different lesion scenarios (Fig. 7), that BLBP^+^ SCRG are the subpopulation of adult WM cells that up-regulate nestin and vimentin in response to lesion. Given that subpopulations of BLBP^+^ SCRG differentially up-regulate SOX2 and/or PCNA during the lesion response, it also appears that different subpopulations of SCRG may be differentially primed (some more mitotic, others more quiescent) to serve progenitor-like and scaffolding roles within the lesioned SC. However, it is also clear that given the devastating long-term effect of SC injury, SCRG are currently not doing enough to promote repair, and may need to be manipulated to facilitate this.

Genome-wide analysis of gene expression in the developing and mature SC has identified a novel progenitor niche in the SC WM, and potential progenitor subtype (SCRG) with a distinct molecular identity. Both neonatal and adult SCRG have a unique expression profile that is similar to NSC, and quite distinct from “WM astrocytes”. Their response to lesion indicates that SCRG may have the capacity to serve as a peripherally-accessible progenitor pool that could be better mobilized in endogenous SC repair strategies, by potentially activating the new candidate NSC regulatory pathways identified here. These data not only illuminate the uniqueness and heterogeneity of SCRG within their niche, but they also highlight how SCRG differ from other CNS progenitors, and identify a set of human disease genes that can now be localized within different CNS progenitor compartments. This SCRG gene expression signature may not only significantly enhances our understanding of NSC regulation, but also serves as an expression map to search for the alternative progenitor-subpopulations in non-ventricular molecular environments throughout the CNS.

## Methods

### Gene Expression Analysis of Allen Spinal Cord Atlas Data

An initial survey of the ASCA (http://mousespinal.brain-map.org/) *ISH* images was performed, and expression patterns binned according to patterns that resembled the expression of known “signature” genes. For SCRG, *Gfap* and *Blbp* (*Fabp7*) were used as the expression template for both neonatal (PND 4) and adult (PND 56) SCRG. Genes expressed by cells arrayed radially and extending processes toward the CC were identified as SCRG genes. Each gene was scored from 0 to 3 at both time points to determine how the expression pattern changed overtime, where *Gfap* and *Blbp* were considered 100% expressed in all SCRG: (0) Genes not found in SCRG; (1) Genes expressed in 1–20% of the SCRG; (2) Genes expressed in 80–20% of SCRG; (3) Genes that were highly expressed and present in >80% of SCRG (see Fig. S1 for examples). The genes expressed in the CC were scored similarly. Additionally, we tested both the literature and array expression databases for NSC candidate genes that may have been overlooked in the original survey, and we subsequently scored each for enrichment in SC progenitors. Once confirmed, these additional genes were added to the respective datasets.

### AGEA Analysis of the adult Mouse subventricular zone

We used the Allen Brain Atlas AGEA tool to test for progenitor zone expression of the SVZ. To do this, we sampled gene expression bilaterally at three different locations on the rostro-caudal SVZ axis. To remove background (non-neurogenic) expression, adjacent regions of corpus callosum and GM were sampled using AGEA to generate an expression profile which was subtracted from the SVZ list. All candidate genes were then verified visually to establish their enrichment in the SVZ alone (i.e. some original candidates were excluded as they came up in the choroid plexus upon visual confirmation).

### Comparison of Progenitor Zone Expression Profiling

The expression pattern of SCRG genes was compared between the neonatal and adult gene lists using the pangloss Venn diagram generator (www.pangloss.com/seidel/protocols/venn.cgi). This program was also used to compare the entire SCRG gene list to the CC and SVZ data sets.

### Gene Ontology Analysis

To analyze the functional categories represented in SCRG, the Database for Annotation, Visualization and Integrated Discovery (DAVID: http://david.abcc.ncifcrf.gov/) was used. The official gene symbols of the SCRG data set were submitted as a *Mus Musculus* gene list and the ‘Functional Annotation Clustering’ table was examined for ontologies that corresponded to the listed functional categories (Table 1). The genes from the corresponding ontologies were pooled and listed in the appropriate category, and the percent was determined by comparing the number of genes in each ontology to the total number of SCRG genes (122).

### Experimental Autoimmune Encephalomyelitis

EAE was performed using a MOG_35–55_-induced paradigm as previously described [32]. Briefly, mice were immunized with 100 µg MOG (35–55) peptide (MEVGWYRSPFSRVVHLYRNGK) in complete Freund's adjuvant containing 200 µg heat-killed *Mycobacterium tuberculosis H37RA* by subcutaneous injection over 3 sites on the flank at day 0, with additional intraperitoneal injections of Pertussis toxin (200 ng in sterile saline) on days 0 and 2. Phenotypic assessment was performed daily following the scoring: (0) no signs of disease; (1) limp tail or hindlimb weakness; (2) limp tail and hindlimb weakness; (3) hind limb paralysis. Mice were sacrificed at 18–21 days after induction (Score 2–3, peak disease) and tissue prepared as below.

### Spinal Cord Lesion

A SC compression injury was performed on male C57Bl6 mice using a pair of forceps with custom made spacers to limit the closure to a gap 0.35 mm (for details see [33]. This injury spares a significant amount of tissue and leads to moderate to mild motor deficits. Mice were sacrificed 2 weeks after lesion and sections generated as outlined below, to test for gene and protein expression at and adjacent to the lesion site.

### Tissue Preparation

In accordance with the Canadian Animal Care Committee standards, adult and neonatal mice were anesthetized with 25 mg/ml Xylaket: [120 mg/kg ketamine HCL (MTC Pharmaceuticals, Cambridge, ON) and 12 mg/kg xylazine (Bayer, Tarrytown, NY), 15% ethanol, 0.55% NaCl], perfused transcardially with 0.1 M phosphate buffered saline (PBS) and 4% paraformaldehyde (PFA) in PBS. Whole vertebral columns were extracted and post-fixed overnight in 4% PFA at 4°C. The SC was removed from adult columns, and tissues were cryoprotected in a sucrose gradient before embedded in Tissue-Tek medium (Sakura Finetek, Torrance, CA) and frozen in isopentane on dry ice. Cryosections were prepared (12 µM) and stored at −20°C.

### Antigen Detection by Immunofluorescence

Immunohistochemistry was performed on cross-sections (12 µm) of neonatal (PND 5) or adult (PND 56) SC. Fabp7-EGFP (Blbp; MMRRC #000299-UCD) tissue was post-fixed in 4% PFA, permeabilized with 0.3% Triton-X-100 and blocked in 5% serum for one hour. For nuclear antigens, an antigen-retrieval step of 2 N HCl incubated at 37°C for 30 minutes was performed. Primary antibodies were incubated overnight at 4°C as detailed below. The wild-type SC tissue was processed as previously described [34], [35]: the tissue was post-fixed using 4% PFA, boiled in 0.01 M Citric Acid (pH 9.0) for 20 minutes and permeabilized using 0.1% Triton X-100/PBS for 30 minutes. Before primary antibody incubation the sections were blocked with 4% Normal Serum for 30 minutes at room temperature, and stained overnight at 4°C with: rabbit anti-mouse BLBP (1∶1000, Millipore), mouse anti-rat nestin (1∶200, BD Sciences), rabbit anti-mouse nestin (1∶500, Covance) mouse anti-GFAP (1∶500, Sigma), mouse anti-PCNA (1∶5000; Sigma), mouse anti-SOX2 (1∶1000, R&D Systems) and mouse anti-vimentin (1∶200, Accurate). Secondary antibody (1∶200) incubation with Alexa 350, Alexa 488 or Alexa 594 (Invitrogen) was performed for 60 minutes at room temperature and nuclei were stained with 0.5 µg/ml diaminopyridine imidazole (DAPI). Sections were coverslipped in ProLong Gold (Invitrogen) and visualized using a Zeiss Axioplan II fluorescence/DIC microscope and imaged using a Fluoview FV1000 laser scanning confocal microscope. Images were processed in Volocity (Improvision, Lexington, MA) to generate compressed z-stacks or 3D views. PhotoShop and Illustrator were used to create figure montages (Adobe Systems, San Jose, CA).

### Quantification of SCRG

The images used for quantification were captured at 3 levels of the SC: cervical, thoracic and lumbar. We quantified cells in 3 areas delineated based on anatomical boundaries (see Figure 5A; arrowheads delimitating the areas): dorsal, lateral and ventral. BLBP^+^ cells residing within the 50 µm^2^ area from the edge of the SC cross-section were quantified and characterized based on i) the location of their nuclei (i.e. pial vs. subpial), and ii) their cellular morphology (i.e. radial vs. non-radial). The 50 µm^2^ area for counting was defined in Abode Photoshop CS3 and cell types were quantified using the ImageJ64 cell counter software. Any BLBP^+^ cells that had their soma touching the outer edge of the SC were considered ‘pial’, while soma that were not in direct contact with the edge were considered ‘subpial’ cells. Cells were further classified based on morphology: cells that had a distinct radial process extending from their nucleus were determined to be ‘radial’, all other morphologies (e.g. mulitpolar cells) were grouped as ‘non-radial’. The number of DAPI^+^ cells in each region was also determined using ImageJ 64; the 8-bit grayscale DAPI channel was converted into a binary image, processed using the ‘watershed’ function and DAPI^+^ soma counted using the ‘analyze particles’ plug-in. Differences in percentage of GFP^+^ cells between SC levels (cervical vs thoracic vs lumbar) SC areas (dorsal vs lateral vs ventral) were determined using one-way or two-factor ANOVA followed by Tukey Post Hoc test (SPSS statistic 19). For all statistical analyses, significance was accepted at a *P*-value of less than 0.05. Results were expressed as mean± standard error of the mean (SEM).

## Supporting Information

Figure S1 **Representative Examples of Scoring Strategy Used to Evaluate SCRG Gene Expression.** To evaluate the transcriptional profile of SCRG cells, each gene was assigned a score between 0–3 based on the representation of the ISH signal within the target cell population. Where *Gfap* and *Blbp* were considered 100% expressed in all SCRG, these images provide examples of genes that would be given a score of: (A) 3 = expressed in >80% SCRG, (B) 2 = expressed in 80–20% SCRG, (C) 1 = expressed in 1–20% SCRG, or (D) 0 = not expressed in SCRG. Boxed areas correspond to the magnified images (A′–D′). *ApoE*: Apolipoprotein E; SPARC: secreted protein acidic and rich in cysteine. Scale bars are 100 µm.
(TIF)

Figure S2 **Progenitor Gene Expression in Adult SCRG.** Confocal z-stack images of neural progenitor markers in (D–F) adult SCRG. (A–B) SCRG express nestin robustly in the adult. (B) In adult SCRG, GFP^+^ processes (green) co-express VIM (blue) more abundantly than in neonatal. Their nuclei remain at the sub-pial edge of SC and small subpopulations retain expression of (A, C) SOX2 (blue) and (C) PCNA (red). The reduction in progenitor marker expression from neonatal to adult observed in the CC (Figure 4) is also reflected in SCRG. Arrow: process; double arrowhead: double-positive cell; double arrow: SCRG; dotted line: marginal edge of SC; Vim: Vimentin; BV: blood vessel. Scale bars: 50 µm.
(TIF)

Figure S3 **SCRG Express NSC Proteins in the Neonatal SC and Retain Progenitor-like Morphology and Expression Profile in Adult.** (A, C) Confocal z-stack images of cross-sectioned SC from neonatal (PND 5) mice. (C) BLBP^+^ (green) SCRG processes (double arrow) begin at the marginal edge of the SC and extend through the WM, and subpopulations co-express (A) VIM (red) but rarely NES (green) and (C) their nuclei contain PCNA (red). (B, D) Confocal z-stack images of cross-sectioned SC from adult (PND 75) mice. Although less abundant than in the neonatal SC, the (D) BLBP^+^ (green) processes of adult SCRG also demonstrate enhanced expression of (B) NES (green). Their nuclei remain at the sub-pial edge of SC and small subpopulations retain expression of (D) PCNA (red). (B) A large subpopulation of NES-expressing cells (green) co-express VIM (red). Boxed areas outline magnified regions (A′–D′) of the z-stack that were rotated and tilted on a 3D plane to best highlight the anchored cell soma and processes at the pial boundary. (A″–D″) Schematics of SC cross-section detail the precise expression pattern of the corresponding markers and highlight the shifting cytoarchitecture of the SCRG and CC progenitors of the adult SC. Immuno-positive multipolar cells and BV are included here. Arrow: process; double arrowhead: double-positive cell; double arrow: SCRG; dotted line: marginal edge of SC; BV: blood vessels. Scale bars: 50 µm
(TIF)

Table S1 **List of genes expressed by spinal cord radial glia.** The genes listed here have a distinct SCRG expression pattern, and were identified using the ASCA and testing within the ASCA for known neural stem cell genes. The ISH record for each gene was independently analyzed and assigning a score between 0–3 for both the neonatal and adult time points (for examples see Figure S1). The ontological categories assigned to each of the genes were determined using DAVID.
(XLS)

Table S2 **List of Core Genes Expressed by SCRG, CC and SVZ Cells.** The nine genes that are shared between SCRG, the CC and SVZ progenitor populations are listed here. The gene symbols and official gene names are given along with their Entrez Gene summary, from the NCBI website (National Center for Biotechnology; http://www.ncbi.nlm.nih.gov/entrez). Asterisks mark genes associated with human disease, as listed on OMIM (Online Mendelian Inheritance in Man; http://www.ncbi.nlm.nih.gov/omim) website (excluding *Lxn*; (Muthusamy et al., 2006), with the corresponding disease listed after the gene summary.
(XLS)
