# Supplementary figures and images for: Adult Spinal Cord Radial Glia Display a Unique Progenitor Phenotype
Source: PLoS One. 2011 Sep 12;6(9):e24538. doi: 10.1371/journal.pone.0024538 (PMC3171483; doi:10.1371/journal.pone.0024538)

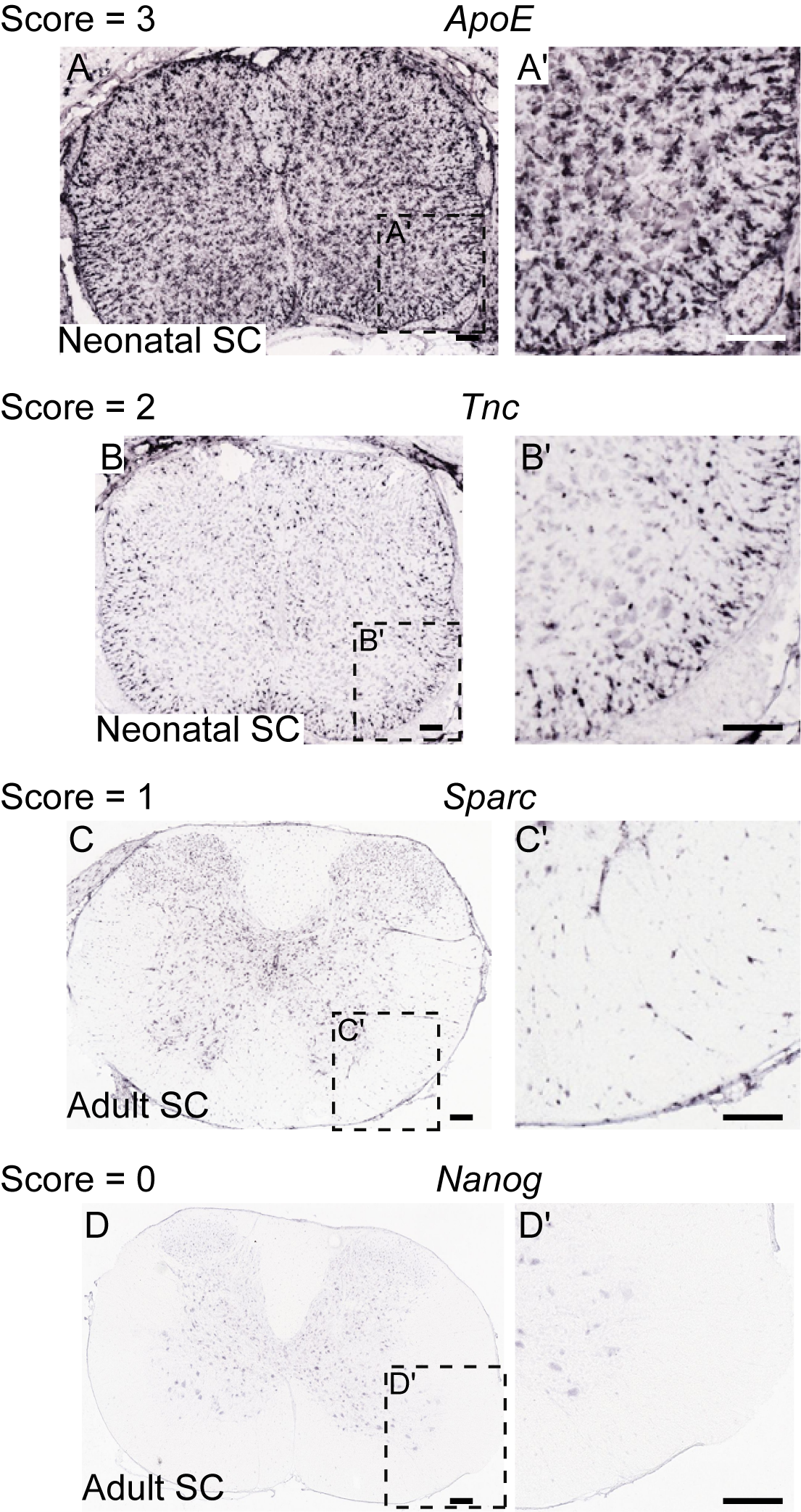

Supplement: Figure S1 — Representative Examples of Scoring Strategy Used to Evaluate SCRG Gene Expression. To evaluate the transcriptional profile of SCRG cells, each gene was assigned a score between 0–3 based on the representation of the ISH signal within the target cell population. Where Gfap and Blbp were considered 100% expressed in all SCRG, these images provide examples of genes that would be given a score of: (A) 3 = expressed in >80% SCRG, (B) 2 = expressed in 80–20% SCRG, (C) 1 = expressed in 1–20% SCRG, or (D) 0 = not expressed in SCRG. Boxed areas correspond to the magnified images (A′–D′). ApoE: Apolipoprotein E; SPARC: secreted protein acidic and rich in cysteine. Scale bars are 100 µm. (TIF) [file pone.0024538.s001.tif]

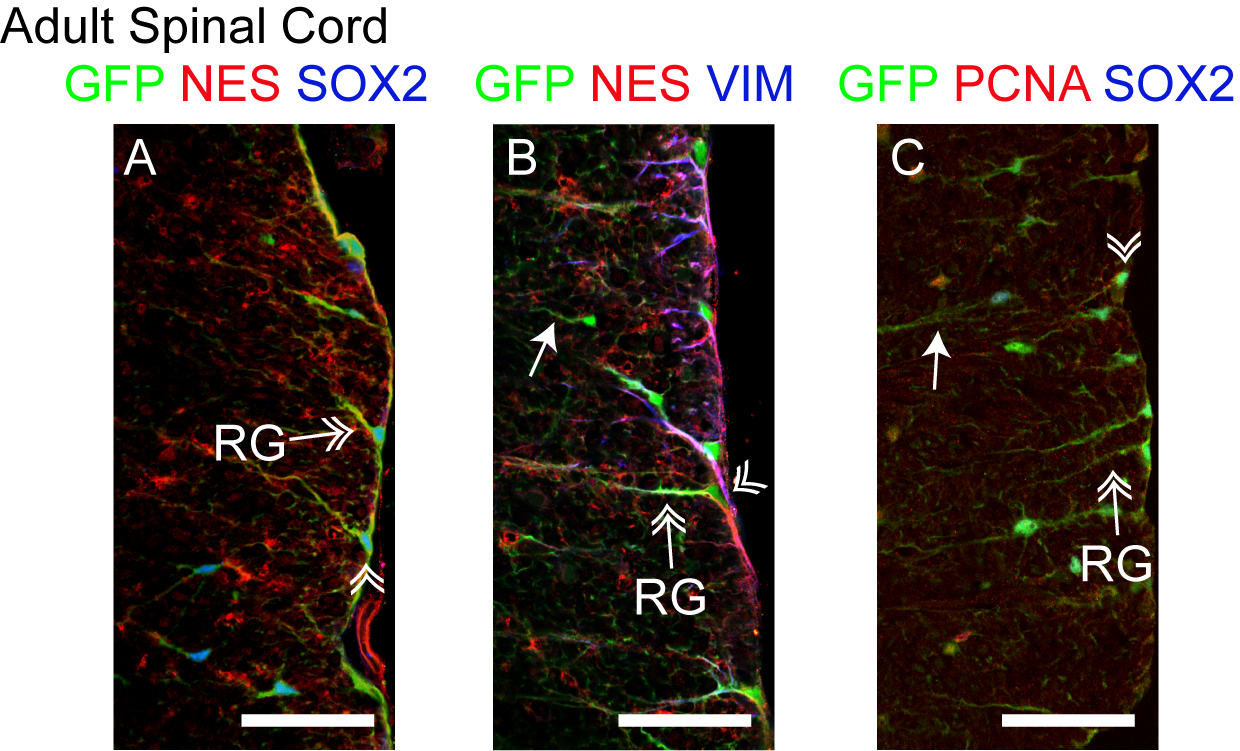

Supplement: Figure S2 — Progenitor Gene Expression in Adult SCRG. Confocal z-stack images of neural progenitor markers in (D–F) adult SCRG. (A–B) SCRG express nestin robustly in the adult. (B) In adult SCRG, GFP+ processes (green) co-express VIM (blue) more abundantly than in neonatal. Their nuclei remain at the sub-pial edge of SC and small subpopulations retain expression of (A, C) SOX2 (blue) and (C) PCNA (red). The reduction in progenitor marker expression from neonatal to adult observed in the CC (Figure 4) is also reflected in SCRG. Arrow: process; double arrowhead: double-positive cell; double arrow: SCRG; dotted line: marginal edge of SC; Vim: Vimentin; BV: blood vessel. Scale bars: 50 µm. (TIF) [file pone.0024538.s002.tif]

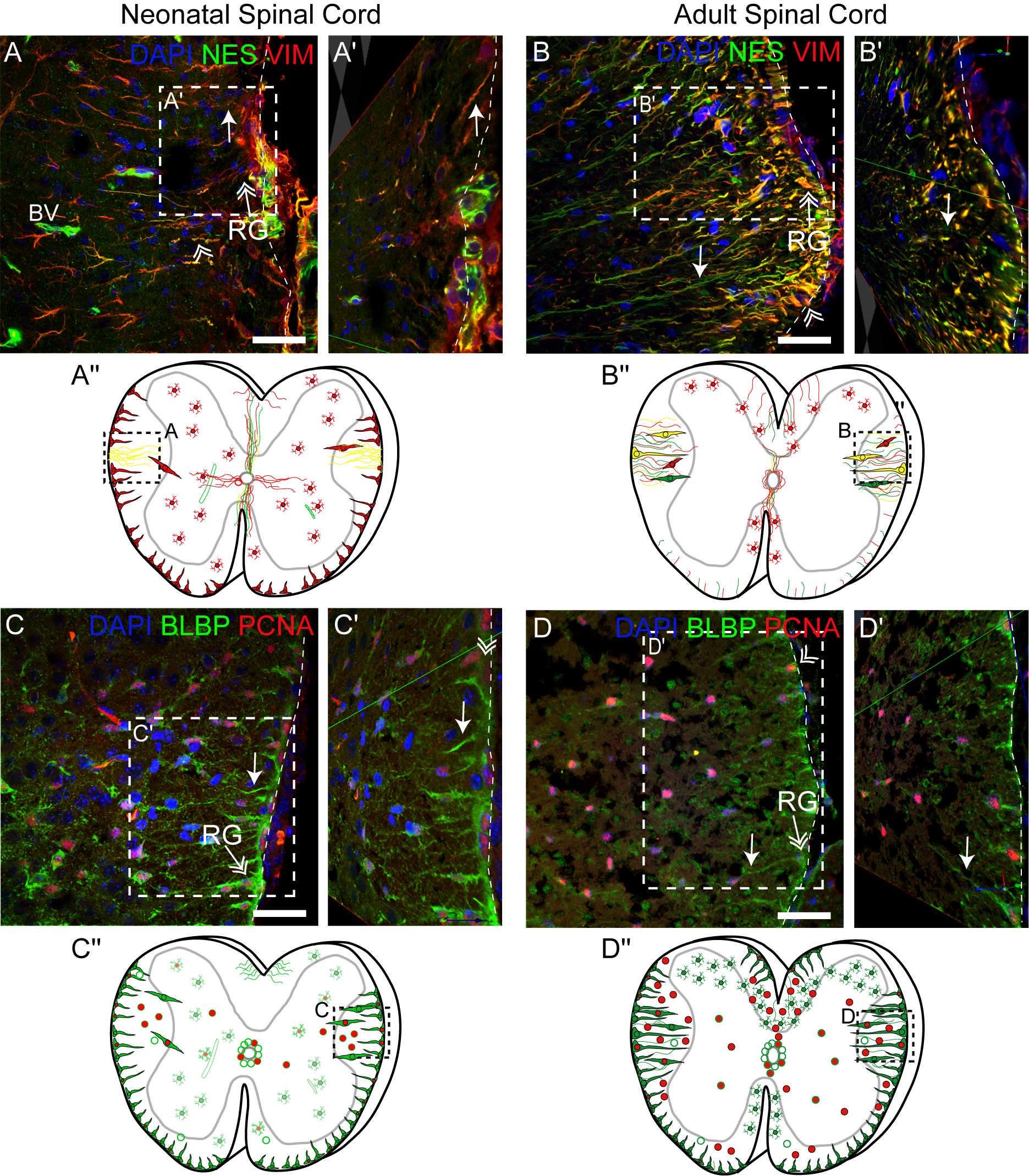

Supplement: Figure S3 — SCRG Express NSC Proteins in the Neonatal SC and Retain Progenitor-like Morphology and Expression Profile in Adult. (A, C) Confocal z-stack images of cross-sectioned SC from neonatal (PND 5) mice. (C) BLBP+ (green) SCRG processes (double arrow) begin at the marginal edge of the SC and extend through the WM, and subpopulations co-express (A) VIM (red) but rarely NES (green) and (C) their nuclei contain PCNA (red). (B, D) Confocal z-stack images of cross-sectioned SC from adult (PND 75) mice. Although less abundant than in the neonatal SC, the (D) BLBP+ (green) processes of adult SCRG also demonstrate enhanced expression of (B) NES (green). Their nuclei remain at the sub-pial edge of SC and small subpopulations retain expression of (D) PCNA (red). (B) A large subpopulation of NES-expressing cells (green) co-express VIM (red). Boxed areas outline magnified regions (A′–D′) of the z-stack that were rotated and tilted on a 3D plane to best highlight the anchored cell soma and processes at the pial boundary. (A″–D″) Schematics of SC cross-section detail the precise expression pattern of the corresponding markers and highlight the shifting cytoarchitecture of the SCRG and CC progenitors of the adult SC. Immuno-positive multipolar cells and BV are included here. Arrow: process; double arrowhead: double-positive cell; double arrow: SCRG; dotted line: marginal edge of SC; BV: blood vessels. Scale bars: 50 µm (TIF) [file pone.0024538.s003.tif]
